# Antitumor Activity of the Cardiac Glycoside αlDiginoside by Modulating Mcl-1 in Human Oral Squamous Cell Carcinoma Cells

**DOI:** 10.3390/ijms21217947

**Published:** 2020-10-26

**Authors:** Jing-Ru Weng, Wei-Yu Lin, Li-Yuan Bai, Jing-Lan Hu, Chia-Hsien Feng

**Affiliations:** 1 Department of Marine Biotechnology and Resources, National Sun Yat-sen University, Kaohsiung 80424, Taiwan; 2 Department of Biotechnology, College of Pharmacy, Kaohsiung Medical University, Kaohsiung 80708, Taiwan; 3 Graduate Institute of Pharmacognosy, College of Pharmacy, Taipei Medical University, Taipei 11042, Taiwan; 4 Department of Pharmacy, Kinmen Hospital, Kinmen 89142, Taiwan; u8557006@gmail.com; 5 Division of Hematology and Oncology, Department of Internal Medicine, China Medical University Hospital, Taichung 40447, Taiwan; lybai6@gmail.com (L.-Y.B.); annavsbelle@yahoo.com.tw (J.-L.H.); 6 College of Medicine, China Medical University, Taichung 40402, Taiwan; 7 Department of Fragrance and Cosmetic Science, College of Pharmacy, Kaohsiung Medical University, Kaohsiung 80708, Taiwan; chfeng@kmu.edu.tw

**Keywords:** cardiac glycoside, αldiginoside, oral squamous cell carcinoma, apoptosis, cell-cycle arrest, Mcl-1

## Abstract

We recently isolated a cardiac glycoside (CG), αldiginoside, from an indigenous plant in Taiwan, which exhibits potent tumor-suppressive efficacy in oral squamous cell carcinoma (OSCC) cell lines (SCC2095 and SCC4, IC_50_ < 0.2 µM; 3-(4,5-dimethylthiazol-2-yl)-2,5-diphenyltetrazolium bromide (MTT) assays). Here, we report that αldiginoside caused Sphase arrest and apoptosis, through the inhibition of a series of signaling pathways, including those mediated by cyclin E, phospho-CDC25C (p-CDC25C), and janus kinase/signal transducer and activator of transcription (JAK/STAT)3. αldiginoside induced apoptosis, as indicated by caspase activation and poly (ADP-ribose) polymerase (PARP) cleavage. Equally important, αldiginoside reduced Mcl-1 expression through protein degradation, and overexpression of Mcl-1 partially protected SCC2095 cells from αldiginoside’s cytotoxicity. Taken together, these data suggest the translational potential of αldiginoside to foster new therapeutic strategies for OSCC treatment.

## 1. Introduction

Oral squamous cell carcinoma (OSCC) is the 11th most common malignancy globally, with over 300,000 new cases and 140,000 deaths reported every year [1]. Tobacco use, alcohol, betel nut, and sexually acquired human papilloma virus are risk factors for OSCC [2]. The standard treatment for OSCC relies on surgery, radiotherapy, chemotherapy, and molecular-targeted therapy. However, the five-year survival rate is only 48%, and recurrence occurs in 20% of patients within two to three years [3]. Therefore, to provide better treatment for OSCC, new drugs or strategies are urgently needed.

Phytochemicals have served as a source of chemopreventive and chemotherapeutic agents for centuries [4]. For example, in 1992, paclitaxel from *Taxus brevifolia* was approved by the U.S. Food and Drug Administration (FDA) for the treatment of patients with recurrent ovarian cancer and breast cancer [5]. Smith et al. reported that vinorelbine tartrate, a vinca alkaloid, is effective as a first-line treatment for advanced non-small-cell lung cancer [6]. Interestingly, in the past decade, increasing attention has focused on cardiac glycosides (CGs) in cancer treatment [7], as many CGs, including bufalin, ouabain, and digoxin, have been reported to suppress tumor cell growth by inducing apoptosis [8,9,10]. Originally, CGs were used for the treatment of congestive heart failure for their ability to block the activity of Na^+^/K^+^-ATPase, which has been linked to the selective antiproliferative activity of CGs in tumor cells, without affecting normal cell growth [11,12]. Moreover, a number of antitumor targets have been reported for different CGs (oleandrin: nuclear factor-κB (NF-κB) [13] and signal transducer and activator of transcription (STAT)3 [14]; ouabain, digoxin, and proscillaridin: DNA topoisomerase II [15]; divaricoside: myeloid cell leukemia 1 (Mcl-1) [16], suggesting that individual CG might mediate their antitumor effect through distinct mechanisms. From a clinical perspective, a couple of CGs, including Anvirzel and PBI-05204, have been evaluated for their safety and pharmacokinetic profiles in patients with refractory solid tumors in a phase I clinical trial [17,18].

**Figure 1:**
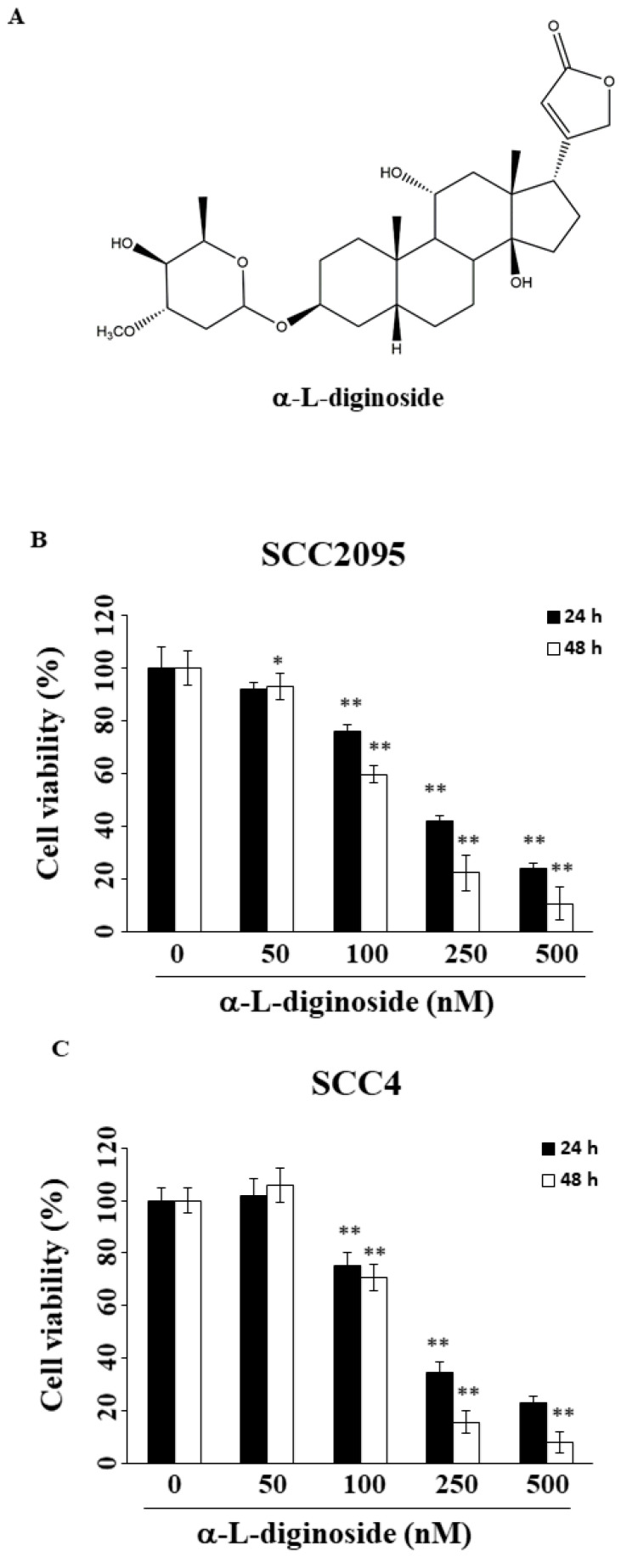
Effect of αldiginoside on the viability of oral cancer cells. (**A**) Chemical structure of αldiginoside. (**B**) SCC2095 and (**C**) SCC4 cells. Cells were treated with αldiginoside in 96-well plates for 24 or 48 h, and cell viability was assessed using 3-(4,5-dimethylthiazol-2-yl)-2,5-diphenyltetrazolium bromide (MTT) assays. *Points* represent means; *bars* represent standard deviation (S.D.) (*n* = 3–6). * *p* < 0.05, ** *p* < 0.01 relative to the control group.

*Strophanthus divaricatus* (Apocynaceae) is an indigenous plant found in Taiwan, from which we have isolated and characterized different CGs with potent antitumor activities [16]. As part of our natural product-based drug development effort, we previously demonstrated the unique ability of one of the CGs isolated from this indigenous plant, divaricoside, to induce apoptosis in OSCC cells [16]. In this study, we further investigated the efficacy and antitumor mechanism of another CG, αldiginoside (structure shown in Figure 1A), in OSCC cells.

## 2. Results

### 2.1. αlDiginoside Inhibits the Proliferation of OSCC Cells

To investigate the antiproliferative activity of αldiginoside, a 3-(4,5-dimethylthiazol-2-yl)-2,5-diphenyltetrazolium bromide (MTT) assay was performed. As shown in Figure 1B,C, αldiginoside inhibited cell growth in a dose- and time-dependent manner in both SCC2095 and SCC4 cells. The IC_50_ values of αldiginoside were 142 nM and 161 nM after 48 h treatment in SCC2095 and SCC4 cells, respectively.

### 2.2. αlDiginoside Induces Cell Cycle Arrest in OSCC Cells

**Figure 2:**
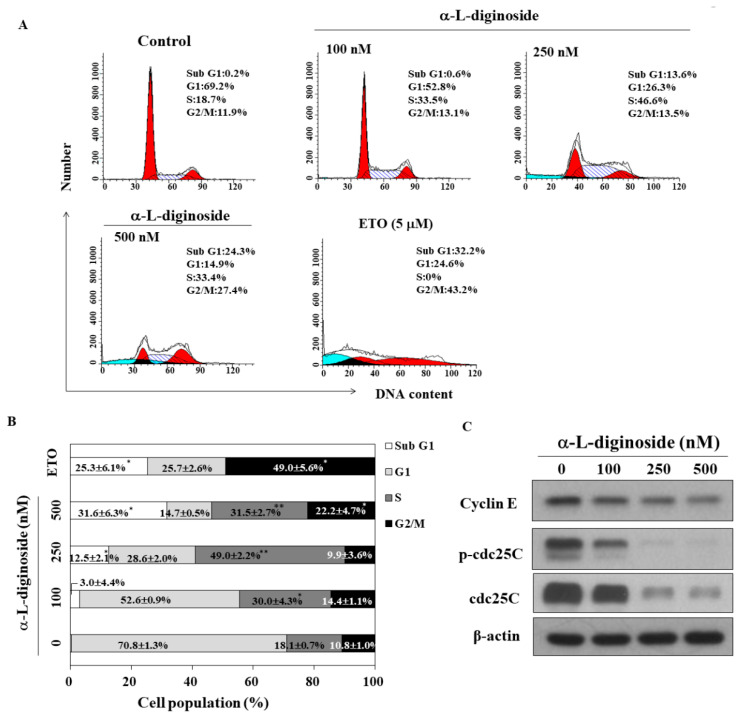
Effect of αldiginoside on cell cycle and cell cycle-regulating proteins. (**A**) Cell cycle analysis showed an increase in the sub G1 and S phase cell populations after treatment of SCC2095 cells with αldiginoside, followed by propidium iodide (PI) staining. Three independent experiments were performed; data are presented in (**B**) as means ± S.D. * *p* < 0.05, ** *p* < 0.01 when compared with the control group. (**C**) Western blotting of lysates of αldiginoside-treated SCC2095 cells showing the phosphorylation and expression of cyclin E and CDC25C. Cells were treated with αldiginoside for 48 h.

Previous studies reported that cardiac glycosides cause cell-cycle arrest in tumor cells [19,20]. We examined the impact of αldiginoside on the cell cycle in SCC2095 cells using flow cytometry. As shown in Figure 2A, treatment with αldiginoside for 48 h induced S phase arrest in SCC2095 cells in a concentration-dependent manner (etoposide was used as a positive control). Compared to the control group, 500 nM αldiginoside increased the percentage of cells in the S phase of the cell cycle from 18.1 ± 0.7 to 31.5 ± 2.7% (Figure 2B). Additionally, the proportion of cells in sub G1 phase was increased in the presence of 250 nM αldiginoside (Figure 2B). At the highest concentration of αldiginoside (500 nM), an increased cell proportion in G2/M arrest was observed (Figure 2B). Western blotting showed that αldiginoside downregulated the expression/phosphorylation of cell cycle-related proteins including cyclin E and phospho-CDC25C (p-CDC25C) (Figure 2C). These results suggested that αldiginoside induced cell cycle arrest at the sub G1 and S phases.

### 2.3. αlDiginoside Induces Apoptosis in OSCC Cells

**Figure 3:**
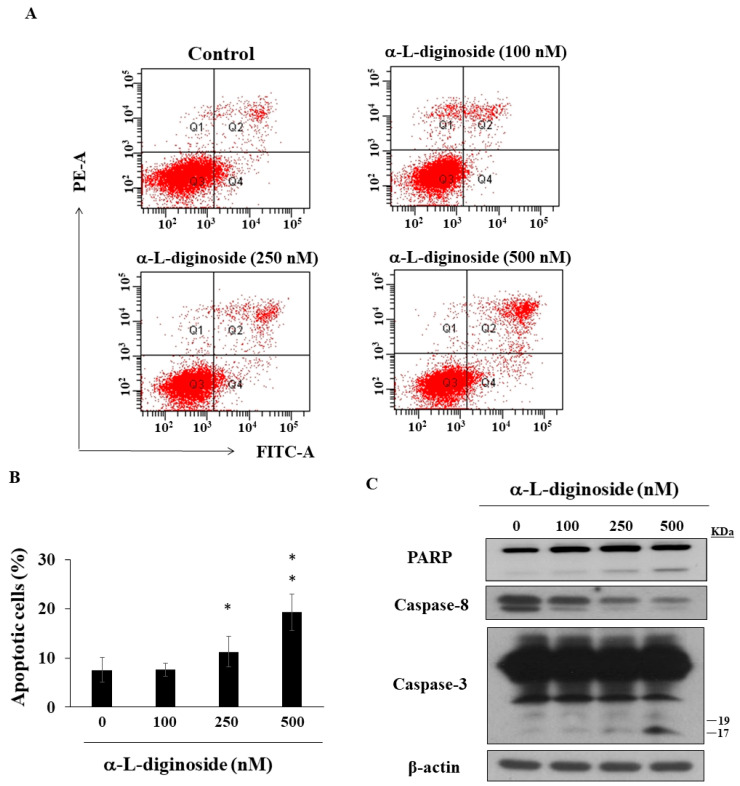
Effect of αldiginoside treatment on apoptosis. (**A**) SCC2095 cells were treated with dimethyl sulfoxide (DMSO) or αldiginoside for 48 h and stained with propidium iodide (PI)/annexin V. (**B**) The percentage of apoptotic cells (Q2 + Q4) is shown. Cells were analyzed using flow cytometry after staining with fluorescein-conjugated annexin V and PI. *Columns* represent means; *bars* represent standard deviations (S.D.) (*n* = 3). * *p* < 0.05, ** *p* < 0.01 when compared with the control group. (**C**) Levels of caspase-3, caspase-8 activation and poly (ADP-ribose) polymerase (PARP) cleavage of αldiginoside-treated SCC2095 cells.

To further confirm whether apoptosis was involved in αldiginoside-induced cell death, SCC2095 cells were treated with αldiginoside for 48 h and subjected to flow cytometry. Propidium iodide (PI)/annexin V staining demonstrated that the proportion of apoptotic cells was increased by treatment with αldiginoside (Figure 3A,B), which was consistent with the observation of sub G1 phase in the cell cycle (Figure 2B). Western blotting showed that αldiginoside increased the expression of cleaved poly (ADP-ribose) polymerase (PARP) and cleaved caspase-3, accompanied by decreased expression of caspase-8 (Figure 3C).

### 2.4. αlDiginoside Modulates JAK/STAT3 Signaling

**Figure 4:**
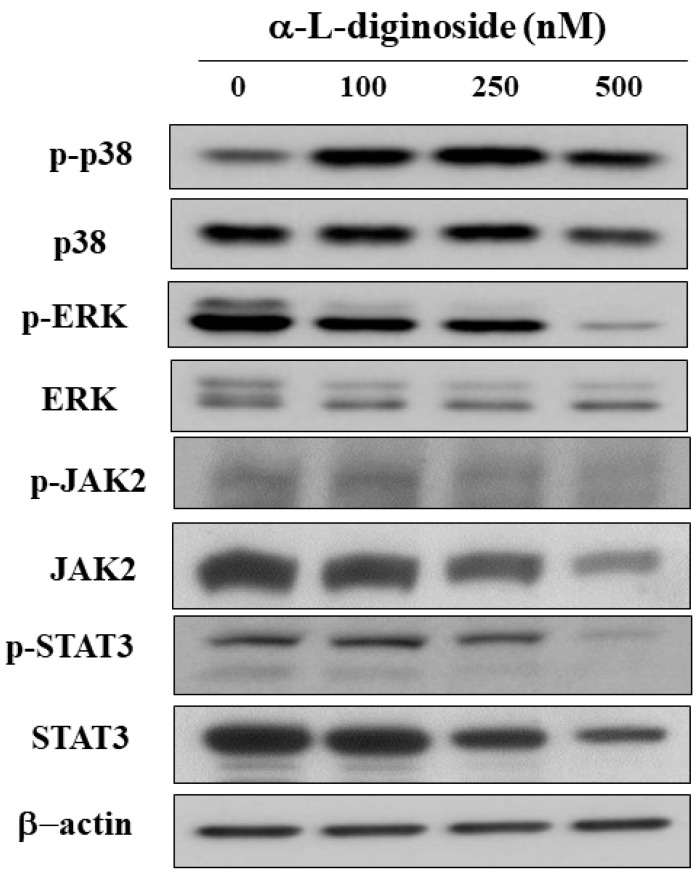
Phosphorylation/expression of p38, extracellular signal-related kinase (ERK), Janus kinase (JAK)2, and signal transducers and activators of transcription (STAT)3 after αldiginoside treatment of SCC2095 cells.

Dysregulation of mitogen-activated protein kinases (MAPKs), and signal transducer and activator of transcription (STAT) is correlated with tumorigenesis of multiple types of cancer, including OSCC [21,22,23]. Western blotting showed that αldiginoside increased the phosphorylation of p38 and increased the phosphorylation of extracellular signal-related kinase (p-ERK). The results also demonstrated that the activation of STAT3 and its upstream target JAK2 was downregulated by αldiginoside in SCC2095 cells (Figure 4).

### 2.5. Mcl-1 Is Involved in αlDiginoside-Mediated Cell Death

**Figure 5:**
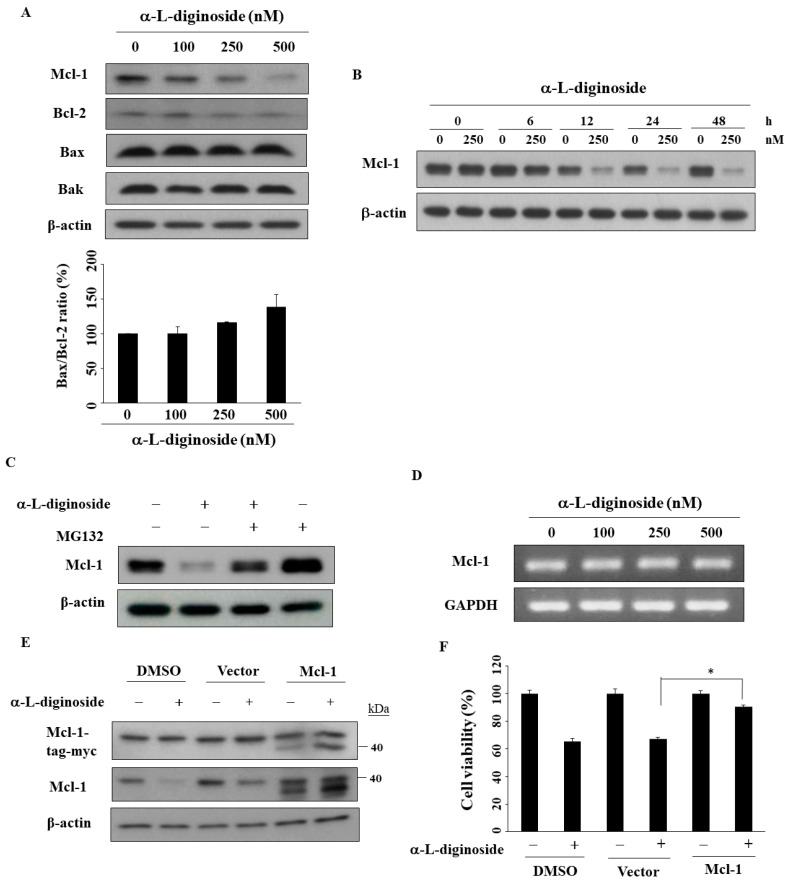
Effect of αldiginoside treatment on expression of the B-cell lymphoma 2 (Bcl-2) family of proteins. (**A**) Upper panel, the expression of myeloid cell leukemia 1 (Mcl-1), Bcl-2, Bcl-2 associated X-protein (Bax), and Bcl-2 homologous antagonist/killer (Bak) after 48 h αldiginoside treatment in SCC2095 cells. Lower panel, the Bax/Bcl-2 ratio. Densitometric quantification of the autoradiograms for Bax and Bcl-2 was performed and calculated. (*n* = 2). (**B**) Time-dependent effect of αldiginoside treatment on the expression of Mcl-1. (**C**) Expression of Mcl-1 with 250 nM αldiginoside alone or in combination with 200 nM MG132. (**D**) *Mcl-1* RNA expression measured by RT-PCR in αldiginoside-treated cells. (**E**) Effect of ectopic Mcl-1 expression after αldiginoside treatment. SCC2095 cells were transfected with control vector or Mcl-1 plasmid for 24 h and treated with 100 nM αldiginoside for 48 h. Whole-cell extracts were subjected to Western blotting. (**F**) Effect of Mcl-1 overexpression on the viability of SCC2095 cells treated with 100 nM αldiginoside for 48 h. After incubation, cells were analyzed using a 3-(4,5-dimethylthiazol-2-yl)-2,5-diphenyltetrazolium bromide (MTT) assay. *Columns* represent means; *bars* represent standard deviations (S.D.) * *p* < 0.05.

Several studies have shown that the antiapoptotic protein Mcl-1 is involved in bufalin- or ouabain-induced apoptosis in cancer cells [10,24]. To evaluate the role of Mcl-1, we examined the effects of αldiginoside on the B-cell lymphoma 2 (Bcl-2) family. As shown in Figure 5A, αldiginoside downregulated the expression of Mcl-1 and Bcl-2 in a dose-dependent manner with no obvious impact on the proapoptotic proteins Bcl-2 associated X-protein (Bax) and Bcl-2 homologous antagonist/killer (Bak) in SCC2095 cells. The ratio of Bax/Bcl-2 expression remained relatively similar after the treatment of αldiginoside (Figure 5A). In a time-course experiment, Mcl-1 expression decreased after treatment with αldiginoside for 12 h in SCC2095 cells (Figure 5B). We obtained two lines of evidence that this Mcl-1 downregulation was mediated through proteasomal degradation. First, MG132, a proteasome inhibitor, abrogated the suppressive effect of αldiginoside on Mcl-1 expression (Figure 5C). Second, RT-PCR analysis showed that *Mcl-1* mRNA expression remained unchanged in αldiginoside-treated cells. Furthermore, we examined the effect of Mcl-1 ectopic expression on the viability of αldiginoside-treated SCC2095 cells (Figure 5D). As shown in Figure 5E,F, Mcl-1 overexpression partially protected cells from αldiginoside-induced cytotoxicity (* *p* < 0.05).

## 3. Discussion

Natural products have served as a rich resource, providing medicinal agents with structural complexity for centuries. Recent studies showed that some CGs, including oleandrin, digoxin, and ouabain, possess antitumor activity [25]. Epidemiological studies have shown lower mortality rates in breast cancer patients receiving CG therapy [26]. Frankel et al. reported that digoxin plus trametinib induces a 20% greater response than trametinib alone in patients with metastatic melanomas [27]. In this study, αldiginoside induced cell-cycle arrest and apoptosis in a dose-dependent manner. In addition, we confirmed that αldiginoside inhibited the proliferation of oral cancer cells by inhibiting Mcl-1.

Takai et al. found that bufalin inhibits cell-cycle arrest at G1 phase, with downregulation of the expression of cyclin A and cyclin D3 in ovarian cancer cells [28]. Ouabain and cinobufagin have been reported to cause S phase arrest and apoptosis in hepatoma cells [29]. It is well known that the cyclin E/cyclin-dependent kinase 2 (CDK2) complex drives DNA replication through the S phase of the cell cycle [30]. In this study, accumulation of αldiginoside-treated SCC2095 cells in S phase was associated with through the inhibition of the expression/phosphorylation of cyclin E, p-CDC25C, and CDC25C. This is in line with an earlier finding that the phosphorylation state of CDC25C plays a key role in regulating M-phase entry in eukaryotic cells [31]. αldiginoside also increased the cell population in G2/M arrest and sub G1 phase (apoptosis) in SCC2095 cells (Figure 2), which is in agreement with other reports [32,33].

In addition to cell cycle arrest, apoptosis is one of the main causes of cell-growth inhibition [34]. Yong et al. reported that oleandrin induces apoptosis through caspase activation in osteosarcoma cells [35]. Evidence shows that CGs induce apoptosis by modulating mitogen-activated protein kinase (MAPK) signaling pathways in cancer cells [36]. For example, strophanthidin downregulated the expression of MAPK kinase (MEK) in hepatoma cells [37]. Yong et al. reported that oleandrin inhibits cell growth by activating p38 in osteosarcoma cells [35]. Our results showed that αldiginoside activated caspase-8 and caspase-3 in SCC2095 cells. Mechanistically, caspase-3, an executioner caspase, could be activated by the extrinsic pathway involving caspase-9 or the intrinsic pathway involving caspase-8 [38], which, in turn, leads to the cleavage of the substrates including PARP, a biomarker of apoptosis [39]. We also observed that the phosphorylation of p38 and ERK were modulated in response to αldiginoside treatment. Recent studies demonstrated that STAT3 signaling is involved in the proliferation, apoptosis, and metastasis of cancer cells [40,41]. For example, Bufalin suppresses cell growth by inhibiting JAK-STAT3 signaling in colon-cancer cells [42], and convallatoxin promotes apoptosis and inhibits angiogenesis through inhibiting JAK2/STAT3 signaling in colon cancer cells [43]. Similarly, we found that αldiginoside decreased the phosphorylation of JAK2 and STAT3 in SCC2095 cells.

The effect of αldiginoside on the expression of the anti-apoptotic protein Mcl-1 provides a potential link to an anti-proliferative mechanism. Overexpression of Mcl-1 is associated with the poor prognosis and cisplatin-resistance in oral cancer, thus representing a promising target for cancer therapy [44]. Multiple lines of evidence have shown that the known primary target of CGs is Na^+^/K^+^-ATPase, and Mcl-1 is most likely one of its downstream signaling components [45,46]. For example, bufalin and ouabain have been shown to downregulate Mcl-1 in lung cancer cells [10,46]. In our study, ectopic expression of Mcl-1 partially rescued αldiginoside-mediated cytotoxicity, which suggests that Mcl-1 expression might be an important marker for the clinical use of αldiginoside.

Our data demonstrated that αldiginoside inhibits the proliferation of OSCC cells with high potency in the nM range. We obtained evidence that the antitumor activity of αldiginoside was attributable, in part to Mcl-1 downregulation, resulting in cell-cycle arrest and apoptosis. These findings suggest the translation potential of αldiginoside to foster new therapeutic strategies for OSCC treatment.

## 4. Materials and Methods

### 4.1. Reagents, Antibodies, and Plasmids

αldiginoside was obtained from *S. divaricatus* collected in Pintung County, Taiwan, in June, 2013 [16]. The 1D and 2D proton nuclear magnetic resonance (NMR) spectral data and mass spectra of αldiginoside were used for the structural identification and purity determination with reference to available literature data (Figures S1–S6) [47]. All other reagents were obtained from Sigma-Aldrich (St. Lois, MO, USA) unless otherwise noted. Test agents were dissolved in dimethyl sulfoxide (DMSO) and added to culture medium with a final DMSO concentration of 0.1%. Antibodies to PARP, ERK, phosphor-ERK^Thr202/Tyr204^, phospho-p38^Thr180/Tyr182^, p38, phospho-JAK2^Tyr1007/Tyr1008^, JAK2, phospho-STAT3^Ser727^, Bak, caspase-3, Mcl-1, cyclin E, phospho-CDC25C^Ser216^, and CDC25C were obtained from Cell Signaling Technologies (Danvers, MA, USA). Antibody to caspase-8 was obtained from Millipore. Antibody to Bcl-2 was purchased from Santa Cruz Biotechnology (Paso Robles, CA, USA). Antibody to Bax was purchased from Abcam (Cambridge, UK). The anti-β-actin was purchased from Sigma-Aldrich (St. Louis, MO, USA). Plasmids expressing Mcl-1 (Myc-DDK-tagged) and empty vector (pCMV6-Entry) were purchased from OriGene Technology (Rockville, MD, USA).

### 4.2. Cell Culture

Both SCC2095 and SCC4 cells (American Type Cell Culture, human tongue primary tumor) were kindly provided by Professor Susan R. Mallery (The Ohio State University). Cells were cultured in DMEM/F12 (Invitrogen, Carlsbad, CA, USA) with 10% heat-inactivated fetal bovine serum (FBS; Gibco, Grand Island, NY, USA), 100 μg/mL streptomycin, and 100 IU/mL penicillin in a humidified incubator with 5% CO_2_ and 95% relative humidity at 37 °C.

### 4.3. Cell Viability Analysis

To assess cell proliferation, cells (5 × 10^3^/200 μL) were seeded in 96-well plates and incubated with 10% FBS medium overnight, and then cells were exposed to DMSO or αldiginoside for 24 h or 48 h [48]. Then, cell viability was determined by 3-(4,5-dimethylthiazol-2-yl)-2,5-diphenyltetrazolium bromide (MTT) assay. In brief, 200 µL of 0.5 mg/mL of MTT in 10% FBS containing DMEM/F12 medium was added and incubated in a 5% CO_2_ incubator at 37 °C for 4 h. After removing the supernatants, 200 µL of DMSO was added to each well to dissolve the crystal formazan dye. The absorbance was measured at 570 nm with a Thermos Scientific MultiSkan GO microplate spectrophotometer (Thermo Fisher Scientific, Waltham, MA, USA).

### 4.4. Western Blot

Cell lysates were prepared with a lysis buffer (50 mM Tris, 150 mM NaCl, 1.0 mM EDTA, 1% sodium deoxycholate, 0.1% Triton X-100, 1 mM PMSF, protease inhibitor cocktail) [48]. Proteins were separated using 8–10% SDS-PAGE gels and transferred to PVDF (Bio-Rad, Herfordshire, UK) membranes. After blocking with skim milk, the transblotted membranes were probed with primary antibodies at 4 °C overnight, followed by secondary antibodies conjugated to horseradish peroxidase at room temperature for 1 h. Protein bands were detected using enhanced chemiluminescence detection kit (Little Chalfont, Buckinghamshire, UK).

### 4.5. Flow Cytometry

Cells (2 × 10^5^/3 mL) were seeded in six-well plates and treated with αldiginoside, etoposide, or DMSO vehicle for 24 h. For cell cycle analysis [16], after being washed twice with ice-cold PBS, cells were fixed in 70% cold ethanol for 4 h at 4 °C. For apoptosis analysis [49], cells were treated with Annexin V-fluorescein isothiocyanate (FITC) (3 μL) and Propidium Iodide (PI) (1 μL) according to the manufacturer’s instructions. Then, cells were counted on a BD FACSAria flow cytometer (Becton Dickinson, Germany).

### 4.6. Transient Transfection for Overexpression

For overexpression of Mcl-1, cells (2 × 10^5^/3 mL) were seeded in six-well plates and transiently transfected with Myc-DDK-tagged plasmids using Fugene HP (Roche, Basel, Switzerland) according to the manufacture’s protocol [48]. Transfected cells were maintained in culture medium for 24 h, and were treated with DMSO or αldiginoside at indicated concentrations for 48 h. Cell viability was assessed by using MTT assays. Proteins were collected for Western blot analysis.

### 4.7. Reverse Transcriptase-PCR (RT-PCR)

Total RNA was extracted from cells treated with Trizol reagent (Invitrogen) and cDNA was prepared by using the RevertAid First strand cDNA Synthesis kit (Ferments, Thermo Scientific) according to the manufacture’s instruction [50]. The primers used were as follows: *Mcl-1* mRNA: (forward) 5′-TGCTTCGGAAACTGGACATC-3′, (reverse) 5′-TAGCCACAAAGGCACCAAAAG-3′ and *GADPH* mRNA: (forward) 5′-AGGTCATCCCTGAGCTGAACGG-3′, (reverse) 5′-CGCCTGCTTCACCACCTTCTTG-3′.

### 4.8. Statistical Analysis

All data are presented as mean ± S.E.M. from three independent experiments. The statistical analyses were determined using Student’s *t* test. Differences were considered significant at * *p* < 0.05 or ** *p* < 0.01.

## Supplementary Materials

The following are available online at https://www.mdpi.com/1422-0067/21/21/7947/s1.

## Abbreviations

FBS	Fetal bovine serum
PBS	Phosphate-buffered saline
MAPKs	Mitogen-activated protein kinases
STAT	Signal transducer and activator of transcription
OSCC	Oral squamous cell carcinoma
CGs	Cardiac glycosides
JAK	Janus kinase
PI	Propidium iodide
PARP	Poly ADP-ribose polymerase
ERK	Extracellular signal-regulated kinases
MEK	MAPK kinase
MTT	3-(4,5-dimethylthiazol-2-yl)-2,5-diphenyltetrazolium bromide
PVDF	Polyvinylidene difluoride
