# Supplementary material for: Antitumor Activity of the Cardiac Glycoside αlDiginoside by Modulating Mcl-1 in Human Oral Squamous Cell Carcinoma Cells
Source: Int J Mol Sci. 2020 Oct 26;21(21):7947. doi: 10.3390/ijms21217947 (PMC7663359; doi:10.3390/ijms21217947)
Supplement: Supplementary file 1 [file ijms-21-07947-s001.pdf]

SUPPORTING INFORMATION

Article

Antitumor Activity of the Cardiac Glycoside α-L-Diginoside by Modulating Mcl-1 in Human Oral Squamous Cell Carcinoma Cells

Jing-Ru Weng [1,2,3,*], Wei-Yu Lin [4], Li-Yuan Bai [5,6], Jing-Lan Hu [5], and Chia-Hsien Feng [7]

[1] Department of Marine Biotechnology and Resources, National Sun Yat-sen University, Kaohsiung 80424, Taiwan
[2] Department of Biotechnology, College of Pharmacy, Kaohsiung Medical University, Kaohsiung 80708, Taiwan
[3] Graduate Institute of Pharmacognosy, College of Pharmacy, Taipei Medical University, Taipei 11042, Taiwan
[4] Department of Pharmacy, Kinmen Hospital, Kinmen 89142, Taiwan; u8557006@gmail.com
[5] Division of Hematology and Oncology, Department of Internal Medicine, China Medical University Hospital, Taichung 40447, Taiwan; lybai6@gmail.com (L.-Y.B.); annavsbelle@yahoo.com.tw (J.-L.H.)
[6] College of Medicine, China Medical University, Taichung 40402, Taiwan
[7] Department of Fragrance and Cosmetic Science, College of Pharmacy, Kaohsiung Medical University, Kaohsiung 80708, Taiwan; chfeng@kmu.edu.tw

Figure		Page
S1	^{1}H NMR spectrum (600 MHz, $CDCl_3$) of α-L-diginoside	3
S2	^{13}C NMR spectrum (150 MHz, $CDCl_3$) of α-L-diginoside	4
S3	HMQC spectrum of α-L-diginoside	5
S4	COSY spectrum of α-L-diginoside	6
S5	HMBC spectrum of α-L-diginoside	7
S6	FAB-MS spectrum of α-L-diginoside	8

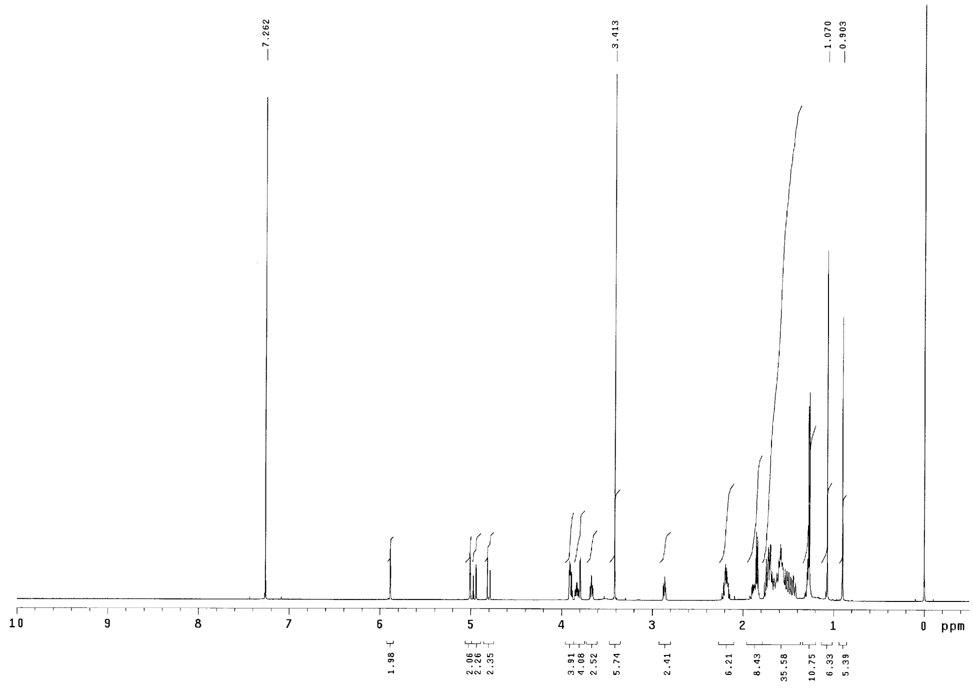

Figure S1. ^{1}H NMR spectrum (600 MHz, $CDCl_3$) of L-diginoside

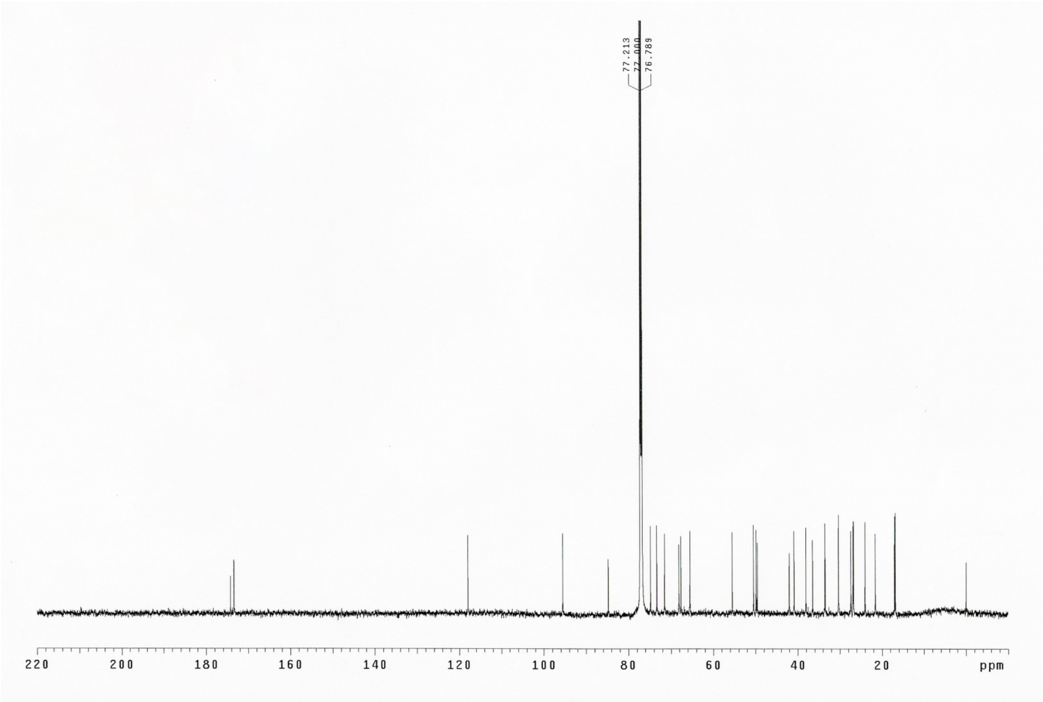

Figure S2. ^{13}C NMR spectrum (150 MHz, $CDCl_3$) of L-diginoside

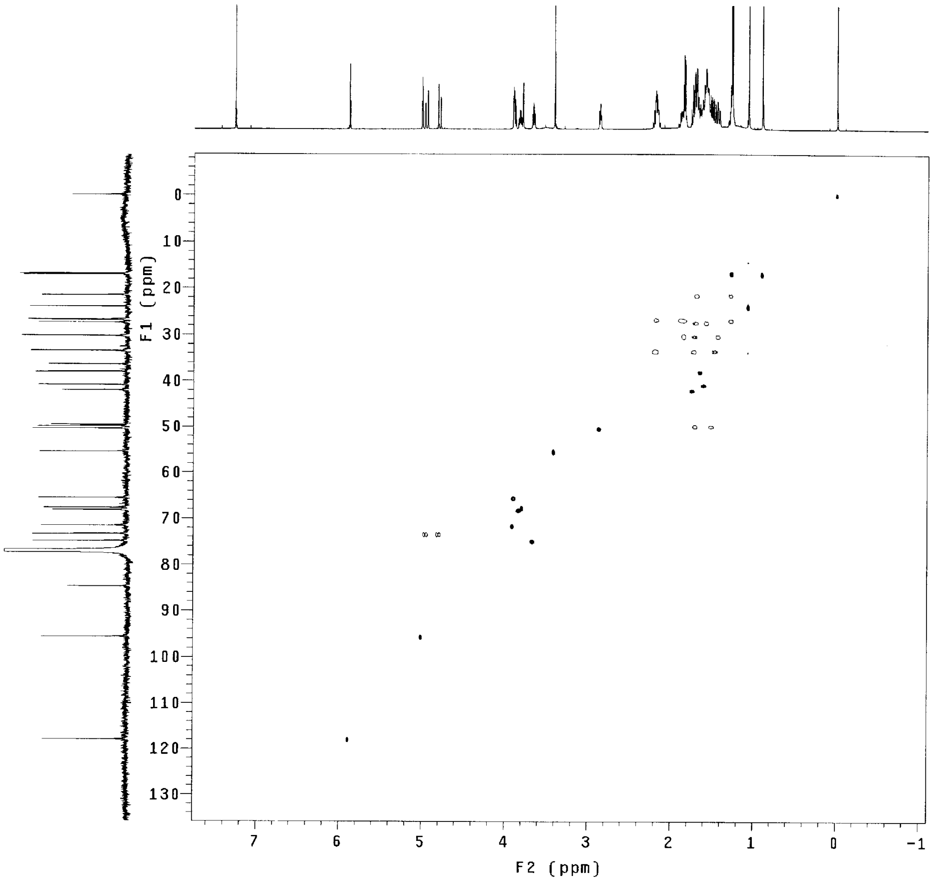

Figure S3. HMQC spectrum of L-diginoside

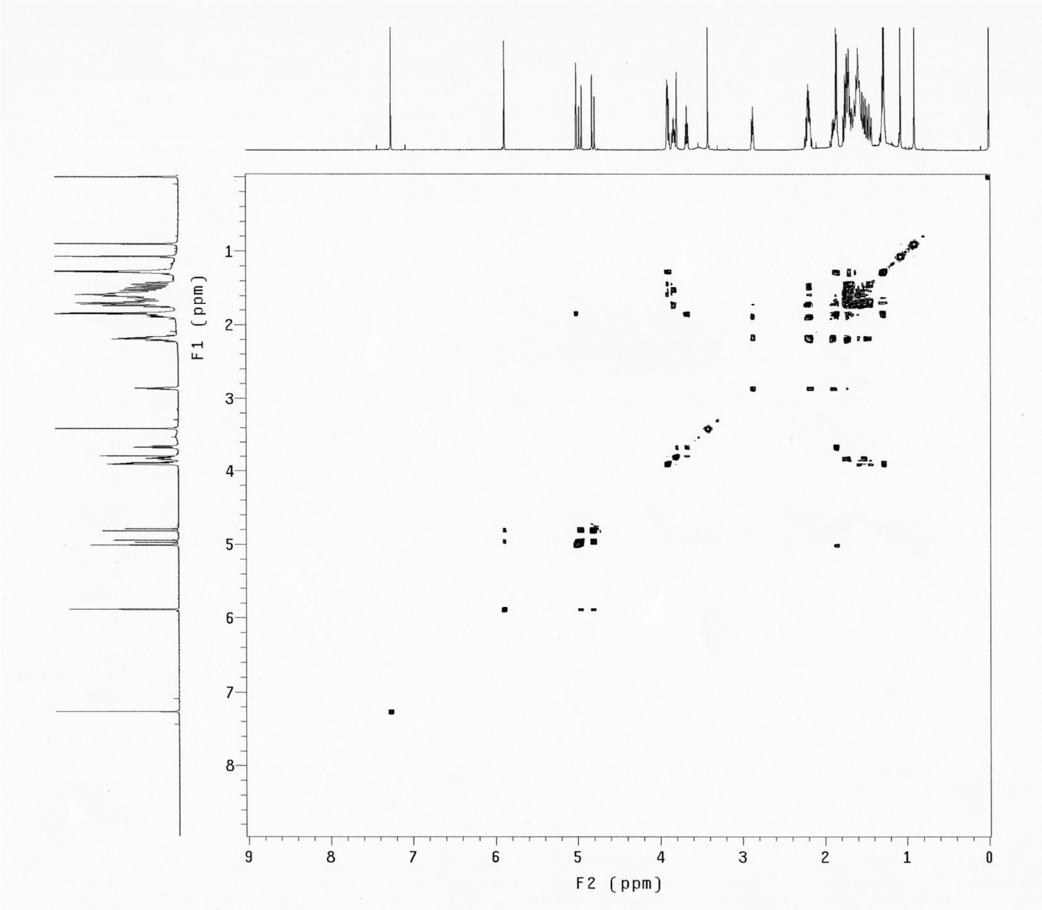

Figure S4. COSY spectrum of L-diginoside

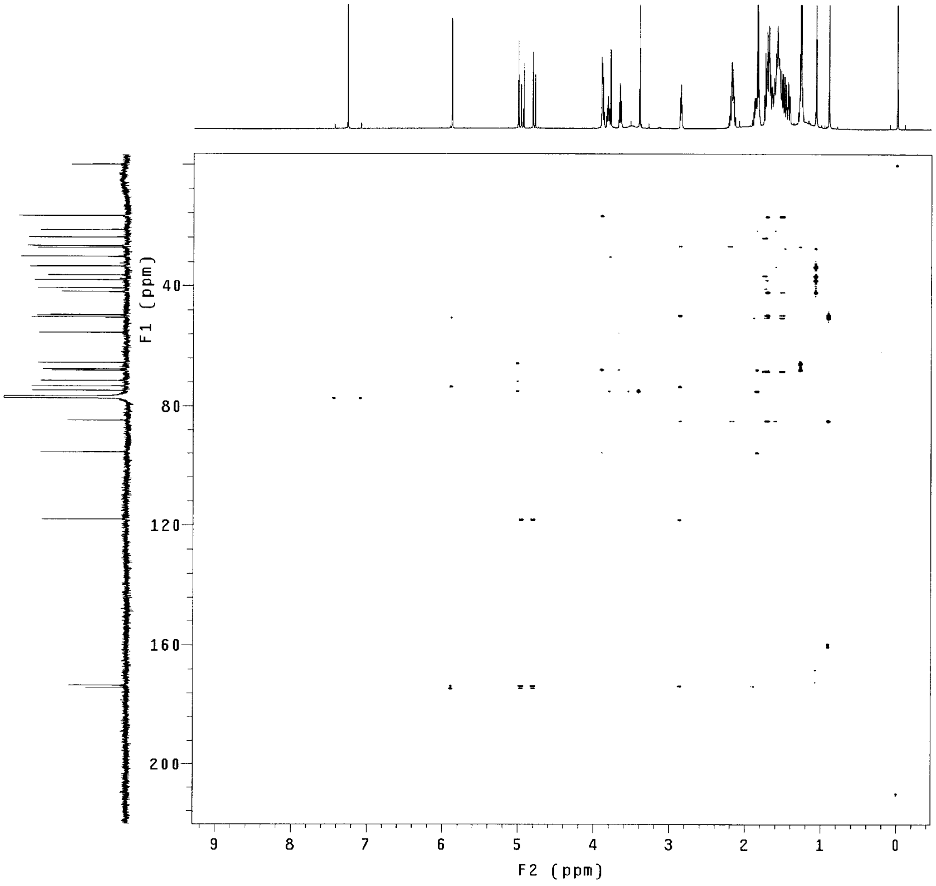

Figure S5. HMBC spectrum of L-diginoside

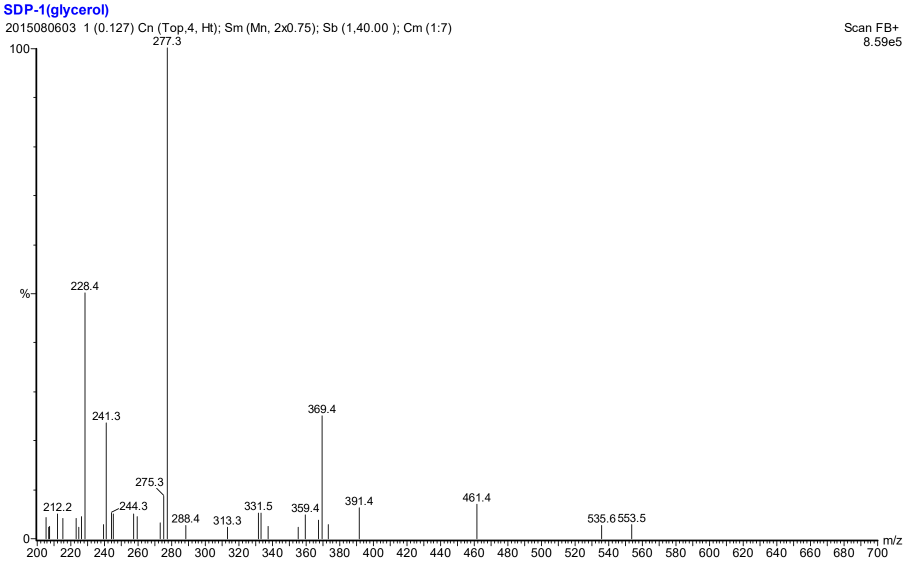

Figure S6. FAB-MS spectrum of L-diginoside
